# Prospective Evaluation of Thermographic Imaging for Early Detection of Pressure Injuries: A Follow‐Up Study

**DOI:** 10.1111/wrr.70119

**Published:** 2025-12-09

**Authors:** Olivia M. Burke, Sophie M. Bilik, Ana Restrepo, Julio Garcia, Zachary J. Frischholz, Ciognay Pire, Monica Sepulveda, Sandra Vera Zuniga, Laura L. Zincone, Gertrudis Gonzalez, Jay Cahill, Siyun Guo, Judith Thichava, Scott A. Elman

**Affiliations:** ^1^ Dr. Phillip Frost Department of Dermatology and Cutaneous Surgery University of Miami Miller School of Medicine Miami Florida USA; ^2^ Inpatient Wound Care Department University of Miami Miller School of Medicine Miami Florida USA; ^3^ Nursing Administration Department University of Miami Miller School of Medicine Miami Florida USA; ^4^ Department of Physical Therapy University of Miami Miller School of Medicine Miami Florida USA

**Keywords:** deep tissue pressure injury (DTPI), diagnostic accuracy, erythema, Fitzpatrick skin type, intensive care unit (ICU), pressure injury, skin of colour, thermography, wound assessment, Wound Scout

## Abstract

Pressure injuries are a major source of morbidity in critically ill patients, and early recognition is particularly difficult in patients with darker skin tones where erythema may be missed. This prospective study evaluated thermographic imaging with the Wound Scout in 32 patients (40 wound encounters) admitted to the ICU. Abnormal temperature, defined as > ±1.1°C, was significantly associated with deep tissue pressure injury (DTPI) diagnosis, with an odds ratio of 6.1 (95% CI 1.2–36.6), sensitivity of 83% and positive predictive value of 74%. Wound Scout changed the diagnosis in 25% of encounters, most often in sacral wounds where visual inspection is limited. Performance was consistent across Fitzpatrick skin types, supporting its utility in skin of colour. Longitudinal data from a small subset (*n* = 10) did not show predictive value for progression. Thermography offers an objective, equitable adjunct to clinical assessment that may improve early detection of pressure injuries.

## 1 Introduction

Pressure injuries are a common and morbid complication in critically ill patients, leading to impaired healing, infection risk, prolonged hospital stays and increased healthcare costs [1, 2, 3]. As we previously described [2], thermographic imaging can detect abnormal temperature differentials that precede visible skin breakdown and is significantly associated with deep tissue pressure injury (DTPI) diagnosis. That retrospective analysis demonstrated both high sensitivity and meaningful clinical impact, including diagnostic changes not captured by routine assessment.

Building on these findings, we conducted a follow‐up study to evaluate thermographic imaging prospectively in an intensive care unit (ICU) cohort. Here, we examine diagnostic accuracy across different skin tones and anatomic locations, assess whether thermography alters clinical diagnosis and explore longitudinal temperature trajectories to determine whether early thermal changes predict wound progression.

## 2 Materials and Methods

### 2.1 Patient Population

A prospective cohort study of patients evaluated at the University of Miami and admitted to the ICU was conducted between July 1, 2024, and July 1, 2025. Eligible participants were adults aged 18 years or older who underwent thermographic assessment with the Wound Scout device during admission. Patients were excluded if they had incomplete medical records, were younger than 18 years, pregnant, or incarcerated. All eligible participants were approached by a member of the study team at the bedside, and written informed consent was obtained before enrolment. The study protocol was approved by the University of Miami Institutional Review Board (Protocol No. 20240712).

### 2.2 Thermographic Assessment

Thermographic imaging was performed using the Wound Scout handheld device at the time of ICU admission and wound nurse consultation. Temperature differentials between wound sites were recorded, with abnormal values defined as greater than ±1.1°C. For each wound, initial and final clinical diagnoses, anatomic location and patient demographics (age, sex, race, ethnicity and Fitzpatrick skin type) were collected. Follow‐up thermographic measurements were obtained at Day 3 and Day 6 when available, as per wound nursing protocol, along with wound progression status. Wounds were categorised as progressing if they deepened, expanded, or converted to DTPI, and stable if no worsening occurred. These data were used to evaluate diagnostic accuracy, subgroup differences and longitudinal wound temperature trajectories.

### 2.3 Statistical Analysis

Age was summarised as mean ± standard deviation, and categorical variables as counts and percentages. Diagnostic accuracy metrics (sensitivity, specificity, positive predictive value [PPV], negative predictive value [NPV]) were calculated for abnormal temperature differentials (> ±1.1°C). Odds ratios (ORs) with 95% confidence intervals (CIs) were derived, and group differences were assessed using chi‐square or Fisher's exact tests. Logistic regression evaluated predictors of DTPI, and generalised estimating equations modelled longitudinal temperature trajectories. Statistical significance was defined as *p* ≤ 0.05.

## 3 Results

**FIGURE 1:**
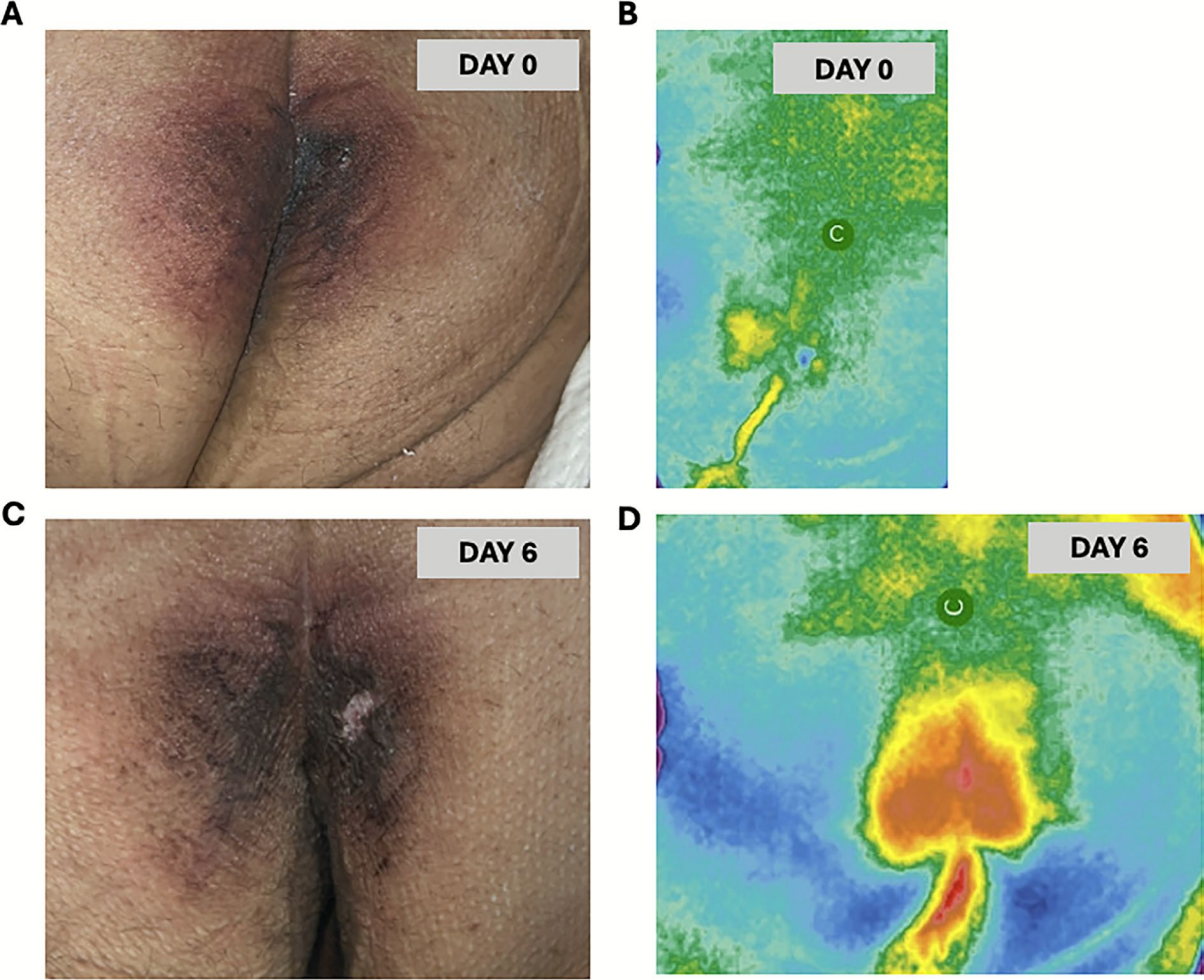
Representative clinical and thermographic progression of a deep‐tissue pressure injury (DTPI). (A, B) Baseline (Day 0) images from a sacral site demonstrate intact skin with subtle discoloration and a corresponding thermogram showing early perfusion asymmetry (cooler zone) before visible breakdown. (C, D) Follow‐up (Day 6) images from the same site show evolution to a visible DTPI with overlying erythema and induration, accompanied by a localised increase in surface temperature (hyperthermic region) on thermography consistent with inflammatory progression. Thermographic scans were obtained using the Wound Scout device; colour gradients represent relative surface temperature (°C), with yellow–red areas indicating warmth and green–blue indicating cooler regions.

A total of 32 patients (40 wound encounters) were included. The mean age was 66.3 ± 11.8 years; 59% were female. Most patients were White (66%), followed by Black or African American (22%) and other races (12%). Forty‐one percent identified as Hispanic/Latino. Representative clinical and thermographic images are shown in Figure 1, illustrating how early perfusion asymmetry on Day 0 corresponded to localised hyperthermia and visible DTPI by Day 6.

### 3.1 Diagnostic Accuracy

Across 40 encounters, abnormal temperature differentials (> ±1.1°C) were detected in 27 cases (67.5%). Among these, 20 (74.1%) were diagnosed as DTPI, compared with 4 of 13 (30.8%) wounds with normal temperatures. This association was significant (OR 6.1, 95% CI 1.2–36.6; *χ* ^2^ = 5.17, *p* = 0.023; Fisher's exact *p* = 0.015), with sensitivity of 83.3%, specificity of 56.2%, PPV of 74.1% and NPV of 69.2% (Table 1). All Stage 1 lesions (*n* = 3) demonstrated abnormal temperature compared with 65% of other wounds, though this difference was not statistically significant (*p* = 0.54).

**TABLE 1:** Results of thermographic imaging in pressure injury detection.

Domain	Metric	Result
Diagnostic accuracy	Association of abnormal temperature (> ±1.1°C) with DTPI	OR = 6.1 (95% CI 1.2–36.6), *p* = 0.015
	Sensitivity	83.3%
	Specificity	56.2%
	Positive predictive value (PPV)	74.1%
	Negative predictive value (NPV)	69.2%
Clinical impact	Overall diagnostic change (any site)	10/40 encounters (25.0%)
	Sacral region	8/21 (38.1%)
	Heel	1/6 (16.7%)
	Foot	1/3 (33.3%)
Skin Tone (Fitzpatrick Subgroup, DTPI only)	I–III	11/13 (84.6%) abnormal
	IV–VI	9/11 (81.8%) abnormal
Ethnicity	Hispanic/Latino	8/22 (36.4%) diagnostic change
	Non‐Hispanic	2/17 (11.8%) diagnostic change

*Note:* Thermographic assessment with the Wound Scout was significantly associated with deep tissue pressure injury (DTPI) diagnosis and led to frequent diagnostic changes across subgroups. Abnormal temperatures were defined as > ±1.1°C. DTPI = deep tissue pressure injury; OR = odds ratio; CI = confidence interval; PPV = positive predictive value; NPV = negative predictive value.

### 3.2 Diagnostic Change

Thermography altered the initial clinical impression in 10 of 40 encounters (25%). Diagnostic change was most frequent in sacral wounds (38%) and the foot (33%), occurred in 17% of heel wounds, and was absent in the hip, ischium and other sites.

### 3.3 Subgroup Analyses by Skin Tone, Race and Ethnicity

Thermographic performance was consistent across skin tones. In DTPI cases, abnormal temperatures were observed in 85% of Fitzpatrick I–III lesions and 82% of Fitzpatrick IV–VI lesions (OR 0.83, 95% CI 0.05–13.5; *p* = 1.0). Diagnostic change rates were likewise similar: 23% in lighter and 28% in darker skin types (OR 1.31, 95% CI 0.31–5.49; *p* = 0.73).

By race, diagnostic changes were most frequent in White patients (39%, 9/23), compared with 11% in Black patients (1/9) and none in Asian (0/3), Hispanic race entries (0/4), or other/unknown categories (0/1). By ethnicity, changes occurred more often among Hispanic/Latino patients (36%, 8/22) compared with non‐Hispanic patients (12%, 2/17).

### 3.4 Longitudinal Follow‐Up

Ten wounds had complete follow‐up to 6 days. Logistic regression showed no association between baseline temperature (OR 0.53, 95% CI 0.11–1.75; *p* = 0.32) or Day 3 change (OR 0.55, 95% CI 0.12–1.84; *p* = 0.36) and progression at Day 6. Generalised estimating equations demonstrated similar temperature trajectories between progressing and stable wounds, with moderate within‐wound correlation (*ρ* ≈ 0.42). Firth logistic regression likewise found no association between baseline temperature, early change, or Fitzpatrick group and progression. Given the small sample (*n* = 10), these longitudinal results remain exploratory.

## 4 Discussion

In this prospective follow‐up study, we evaluated the utility of thermographic imaging with the Wound Scout for early identification of DTPI in critically ill patients. Our design allowed comparison across three steps in the diagnostic pathway: initial clinical assessment, Wound Scout temperature measurement and final diagnosis. A temperature range outside of +1.1°C to −1.1°C was considered abnormal. When patients initially appeared to have only non‐blanchable discoloration but were later diagnosed with DTPI, the Wound Scout was responsible for changing the diagnosis. This occurred in 25% of encounters overall, most commonly in sacral wounds where visual assessment is challenging.

Abnormal temperature strongly predicted DTPI, with nearly six‐fold increased odds of diagnosis compared with normal temperatures. Sensitivity was high (83%), suggesting that Wound Scout can identify most early injuries, while specificity was moderate (56%), reflecting that some non‐DTPI wounds also displayed abnormal temperature. All Stage 1 lesions demonstrated abnormal temperature, highlighting that thermography may detect early physiologic changes even when erythema is the only visible sign. This is particularly important for patients with skin of colour, where erythema may be difficult to appreciate visually. In our cohort, thermographic accuracy was consistent across Fitzpatrick skin types, with abnormal temperatures detected in more than 80% of both lighter and darker skin lesions, and rates of diagnostic change were similar across groups. These findings support the potential role of Wound Scout as an equitable diagnostic adjunct in populations where early visual detection is limited.

Our short ICU follow‐up (6 days) focuses on early detection, which aligns with prior thermography literature showing that the strongest predictive value occurs within the first few days of measurement. Lin et al. demonstrated that the relative temperature between peri‐wound and normal skin on the first day of follow‐up was the most significant predictor of pressure‐injury healing (*r* = 0.687; HR = 8.79, 95% CI 4.53–17.05), whereas later measurements beyond Day 8 were less informative [4]. These findings emphasise that early thermographic changes carry the greatest diagnostic and prognostic value, supporting the design of our short‐term ICU observation window. Similarly, in an ICU cohort of newly admitted patients, Koerner et al. demonstrated that thermal imaging could detect the development of DTPI before visible signs emerged, indicating the value of early surveillance in critical‐care settings [5].

Subgroup analyses further contextualised diagnostic change. Sacral wounds demonstrated the highest reclassification rates, followed by the foot, consistent with the known difficulty of evaluating wounds in high‐pressure, less‐visible sites. By race and ethnicity, diagnostic change was most frequent among White and Hispanic/Latino patients, though interpretation is limited by small subgroup sizes. Still, the trend of more frequent changes in Hispanic/Latino patients raises the possibility that thermography may mitigate disparities in the recognition of early injuries.

We also examined longitudinal data from a small subset of patients (*n* = 10) with repeat measurements. Neither baseline temperature nor early change at Day 3 predicted six‐day progression, and temperature trajectories were similar between wounds that worsened and those that remained stable. While underpowered, these exploratory analyses suggest that static thresholds may be insufficient to predict wound evolution and that larger cohorts are required to determine whether dynamic modelling of temperature changes can forecast progression. Statistical strategies such as mixed‐effects modelling or time‐to‐event analysis could be considered in future studies with larger datasets.

Taken together, our findings indicate that thermographic imaging complements clinical assessment by identifying early physiologic changes that may not be visible on inspection, improving diagnostic accuracy and performing consistently across skin types. Importantly, Wound Scout changed the diagnosis in one‐quarter of encounters, particularly in sacral wounds and abnormal temperature differentials were strongly predictive of DTPI. This technology may be especially valuable in patients with skin of colour, where erythema is often subtle or missed, and a temperature abnormality provides an objective marker. Larger studies are warranted to validate longitudinal predictive value, refine thresholds for progression and evaluate integration of thermography into clinical workflows.

## Conflicts of Interest

The authors declare no conflicts of interest.

## Data Availability

The data that support the findings of this study are available on request from the corresponding author. The data are not publicly available due to privacy or ethical restrictions.
